# New multi-scale perspectives on the stromatolites of Shark Bay, Western Australia

**DOI:** 10.1038/srep20557

**Published:** 2016-02-03

**Authors:** E. P. Suosaari, R. P. Reid, P. E. Playford, J. S. Foster, J. F. Stolz, G. Casaburi, P. D. Hagan, V. Chirayath, I. G. Macintyre, N. J. Planavsky, G. P. Eberli

**Affiliations:** 1 Rosenstiel School of Marine and Atmospheric Science, University of Miami, Miami, Florida, 33158, USA; 2 Bush Heritage Australia, Melbourne, Victoria, 3000, Australia; 3 Geological Survey of Western Australia, Perth, 6004, Western Australia; 4 Department of Microbiology and Cell Science, University of Florida, Space Life Science Lab, Merritt Island, Florida, 32953, USA; 5 Department of Biological Sciences, Duquesne University, Pittsburgh, Pennsylvania, 15282, USA; 6 NASA Ames Research Center, Moffett Field, CA, 94035, USA; 7 National Museum of Natural History, Smithsonian Institution, Washington, DC 20013, USA; 8 Department of Geology and Geophysics, Yale University, New Haven, CT 06520, USA

## Abstract

A recent field-intensive program in Shark Bay, Western Australia provides new multi-scale perspectives on the world’s most extensive modern stromatolite system. Mapping revealed a unique geographic distribution of morphologically distinct stromatolite structures, many of them previously undocumented. These distinctive structures combined with characteristic shelf physiography define eight ‘Stromatolite Provinces’. Morphological and molecular studies of microbial mat composition resulted in a revised growth model where coccoid cyanobacteria predominate in mat communities forming lithified discrete stromatolite buildups. This contradicts traditional views that stromatolites with the best lamination in Hamelin Pool are formed by filamentous cyanobacterial mats. Finally, analysis of internal fabrics of stromatolites revealed pervasive precipitation of microcrystalline carbonate (i.e. micrite) in microbial mats forming framework and cement that may be analogous to the micritic microstructures typical of Precambrian stromatolites. These discoveries represent fundamental advances in our knowledge of the Shark Bay microbial system, laying a foundation for detailed studies of stromatolite morphogenesis that will advance our understanding of benthic ecosystems on the early Earth.

Dominating the fossil record for 80% of Earth history, microbial reefs known as stromatolites are among the most widespread and easily recognized components of Precambrian carbonate platforms^1^. Despite over 100 years of research, the origin and significance of these structures, and indeed the very definition of stromatolites, are still disputed. In this paper, the term ‘stromatolite’ is used for all organo-sedimentary buildups formed by the sediment trapping, binding and/or carbonate precipitating activities of microorganisms, as defined by Awramik *et al.* 1976^2^. This definition maintains traditional terminology, where all microbial structures in Hamelin Pool are referred to as ‘stromatolites’, regardless of degree of lamination^3^.

The first known modern stromatolites with sizes and shapes equivalent to Precambrian forms were discovered in Hamelin Pool, a hypersaline embayment in Shark Bay, by Playford in the 1950’s^3^. For many years following their discovery, Hamelin Pool stromatolites were the primary basis of comparison for fossil examples and these structures have had a profound impact on stromatolite research^4^,^5^. With an area of about 1400 km^2^ and a shoreline of about 135 km almost entirely populated by microbial mats and stromatolites, Hamelin Pool is the largest modern stromatolite system in the world. Previous studies indicate stromatolites have been forming in Hamelin Pool for the past 2000 years, with two growth phases. The first growth phase was 2000–1100 years BP, when relative sea level was approximately 1.5 m higher than present. The second growth phase was 900 years BP to present, at present sea levels^6^,^7^.

Here, we present results from a recent field program in Hamelin Pool that was conducted over a three year period (2012–2014). Extensive in-water observations and sampling throughout the pool (Supplementary Fig. S1), complemented by high-resolution remotely captured imagery and molecular analyses, provide fundamentally new perspectives on stromatolite growth and distribution. From macro- to microscales, our results contrast with traditional models of distribution, growth, and accretion of stromatolites in Hamelin Pool.

## Results and Discussion

### Environmental setting

**Figure 1:**
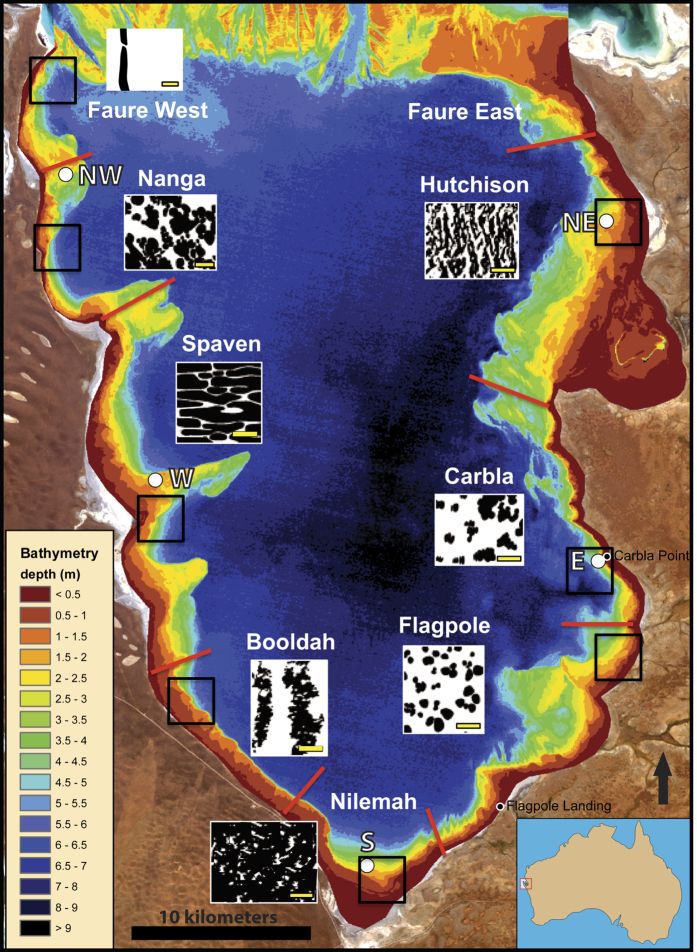
Map depicting bathymetry of Hamelin Pool Provinces. Province boundaries (red lines extending from shore) and characteristic structures in each Province (schematic cartoons) are shown. Cartoons depict structures (discrete buildups or sheets) in black with surrounding sediments in white; yellow scale Bar = 1 m. White circles indicate positions of environmental data loggers (Supplementary Table S1). Detailed bathymetry and imagery within boxes outlined in black are shown in Fig. 2 and Supplemental Fig. S2. Structures of East Faure Province are similar to those in West Faure. Map created in ArcGIS; Basemap sources: Esri, DigitalGlobe, Earthstar Geographics, CNES/Airbus DS, GeoEye, USDA FSA, USGS, Getmapping, Aerogrid, IGN, IGP, swisstopo, and the GIS User Community.

Depth soundings were combined with satellite imagery to produce a detailed bathymetry map of Hamelin Pool (Fig. 1; see methods). Shelf morphology is highly variable. The western margin of the pool is characterized by three major promontories and a narrow shelf. The eastern shelf is variable, forming a broad, gently sloping ramp in the north, a small shelf dropping swiftly into a subtidal zone in the central section, and transitioning back to a gently sloping ramp in the southeast. The southern embayment is a large, gently sloping ramp. Maximum water depth in the basin is 11 m. This high-resolution analysis provides new insight into the submerged terrain of Hamelin Pool and helps to revise previous bathymetry models^6^,^7^,^8^,^9^, which have insufficient resolution to explore physiography in detail, or inaccurate derived depths due to spectral variance between different bottom types^10^.

Environmental parameters of salinity, temperature and pressure (i.e. tidal data), as logged at five key locations around the margin of Hamelin Pool (Fig. 1; see methods), indicated that Hamelin Pool experienced high ranges of salinity and temperature (Supplementary Table S1). With generally hypersaline conditions, recorded salinities ranged between 15.8 and 88.1%, averaging 66%, including higher average salinities in Austral winter months than in summer months. Due to relatively shallow depths (<5 m on shelves and <11 m in the basin), recorded water temperatures closely mirror air temperatures, ranging between 11^o^ and 33 ^o^C and averaging 22 ^o^C. Tidal variation is sinuous, ranging up to 2 m over the course of the year, with higher highs and lows in the winter months and lower highs and lows in the summer months. Daily tidal range is ~1 m depending on meteorological and astronomical input. Current meters detected highest average currents and maximum velocities near the Faure Sill, dampening southward toward Nilemah embayment.

### The Stromatolite Provinces of Hamelin Pool

**Figure 2:**
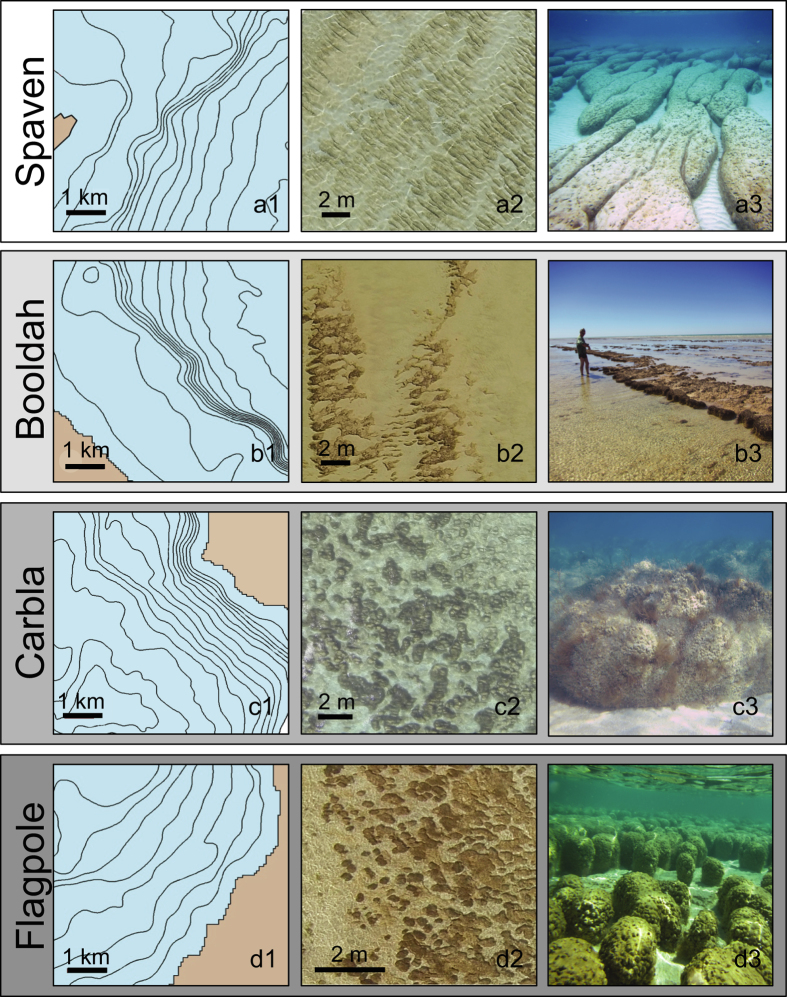
Characteristic features of select Provinces. Column 1 shows (0.5 m) depth contours extracted from the bathymetry map (Fig. 1, boxes outlined in black); see text for discussion of physiography. Representative structures for each Province, shown in columns 2 and 3, are as follows: Spaven – elongate nested stromatolites; Booldah – bands of seif stromatolites; Carbla – massive composite and segmented structures with abundant macroalgae; Flagpole – classic individual and merged columns. Column 2 shows an example of sub-centimeter scale 2D imagery collected via UAV platform and processed using Fluid Lensing (see methods). Field photos in column 3 show characteristic structures with relief as follows: **a3** ~ 40 cm; **b3** 20–30 cm; **c3** ~ 1 m; **d3** 30–40 cm. See also Supplementary Fig. S2 for additional provinces.

Our intensive mapping program revealed a geographic distribution of morphologically distinct stromatolite structures around the margins of Hamelin Pool. This geographic zonation allowed the differentiation of eight ‘Stromatolite Provinces’, each with distinct structures and unique shelf physiography (Figs 1 and 2 and Supplementary Fig. S2).

Some of the distinctive stromatolite morphologies in the various Provinces have not previously been reported in Hamelin Pool. Field observations for early studies^8^,^9^,^10^,^11^ were typically concentrated near the well-known locations of Carbla Point and Flagpole Landing and occasionally included Nilemah Embayment and Booldah Well. In addition, previous research^8^,^9^,^10^,^11^,^12^ has classified stromatolites based on morphology of surface microbial mats and degree of lamination, recognizing pustular mat stromatolites (with irregular surfaces and unlaminated internal fabrics), smooth mat stromatolites (with smooth surfaces and well laminated internal fabrics) and colloform mat stromatolites (with colloform surfaces and moderate lamination). Following this traditional approach, Jahnert and Collins^6^,^7^,^8^,^9^,^10^,^11^,^12^,^13^ produced detailed facies maps showing the distribution of pustular, smooth, and colloform structures plus a new form termed cerebroid, based mainly on interpretation of aerial orthophotos, with ground truthing concentrated in the southeastern margin of the pool^6^.

Classification of stromatolites based on surface mat type provides limited information regarding the underlying structure as i) stromatolites are typically complex structures built by more than one mat type^6^,^7^,^8^ and ii) many stromatolites are relict forms that are not currently accreting, but may be colonized by living microbial mats. Therefore, we adopted a mapping approach that focused on three dimensional structure, rather than surface morphology. As such, we mapped sheets, which are stratiform deposits of poorly lithified, laterally extensive microbial mats^8^, and a variety of discrete lithified microbial buildups. These structures, together with associated sediments and pavements, were mapped in detail based on extensive ground truth observations throughout the Pool (Supplementary Fig. S1). Structures and physiography defining the eight Stromatolite Provinces are as follows (progressing counterclockwise around the pool from the northwest):

#### Faure Province

At the north end of Hamelin Pool, on both sides nearest to the Faure Sill, the margins are characterized by ribbon reefs composed mainly of rock rubble, eroded stromatolites, and microbial pavement, all blanketed in macroalgae. On the western margin, the shelf forms a narrow gently sloping ramp with a very narrow intertidal zone. On the eastern margin, the shelf forms a broader gently sloping ramp with a wider intertidal zone (Fig. 1 and Supplementary Fig. S2). With proximity to open marine conditions north of the Faure Sill, Faure Province hosts healthy sea grass beds and a high diversity of benthic species, including abundant mussel beds and sponges.

#### Nanga Province

In the northwest, pool margins are characterized by massive tabular structures composed of merged columnar heads with abundant macroalgae. These stromatolites occur in a large bight that is flanked by promontories to the north and south. The bight features a narrow, gently sloping ramp, and narrow intertidal zone (Fig. 1 and Supplementary Fig. S2).

#### Spaven Province

In the west, the pool margin is characterized by the extensive occurrence of subtidal elongate nested stromatolites, with direction of elongation perpendicular to shore. These nested structures form to the south of each of three promontories that dominate shelf physiography (Figs 1 and 2).

#### Booldah Province

In the southwest, the pool margin is characterized by a prominent pattern of stromatolites forming north-south bands (seif structures) tens of meters in length, parallel to prevailing wind direction. These seif stromatolites are composed of merged heads with scalloped lobes perpendicular to shore, in the direction of wave translation. Physiography is characterized by a narrow, shallow shelf that drops abruptly into the basin (Figs 1 and 2).

#### Nilemah Province

In the south, the pool margin is characterized by extensive tidal flat deposits of unlithified to poorly-lithified sheets of microbial mats. Shelf physiography is a low-grade ramp that slopes into the basin (Fig. 1 and Supplementary Fig. S2).

#### Flagpole Province

In the southeast, the pool margin is characterized by individual and merged columnar stromatolites. These stromatolites occur seaward of two main headlands and on a gradually sloping ramp that extends nearly seven kilometers in some areas (Figs 1 and 2). Flagpole Landing, within this province, is a focal point for previous studies^4^,^5^,^6^,^7^,^8^,^13^,^14^.

#### Carbla Province

On the eastern margin, the pool margin is characterized by massive stromatolites that are composite segmented structures heavily colonized by the macroalga *Acetabularia*. Shelf physiography is delineated by a narrow intertidal shelf, with water depths <1 m, followed by an abrupt drop to a subtidal ramp (Figs 1 and 2). The massive stromatolites occur on the ramp at depths of 2–4 m. The intertidal shallow shelf is home to the classic relict columnar stromatolites commonly photographed at Carbla Point, which were stranded by falling sea level approximately 1000 years ago^6^.

#### Hutchinson Province

In the northeast, the pool is characterized by elongate clustered stromatolites with elongation perpendicular to shore. The shelf is a broad, gently sloping ramp with abundant live coquina beds (Fig. 1 and Supplementary Fig. S2).

Kilometer-scale areas chosen to depict key attributes of each province were imaged at high-resolution in April 2014 using a quadcopter; frames from this imagery are shown in Fig. 2 and Supplementary Fig. S2. Over 648 km were flown, resulting in approximately 14 square kilometers of imagery with sub-centimeter resolution. This collection is to date, the largest and highest resolution set of ortho-rectified shallow water imagery (Chirayath pers. comm.).

### Microbial mat communities: a revised model

**Figure 3:**
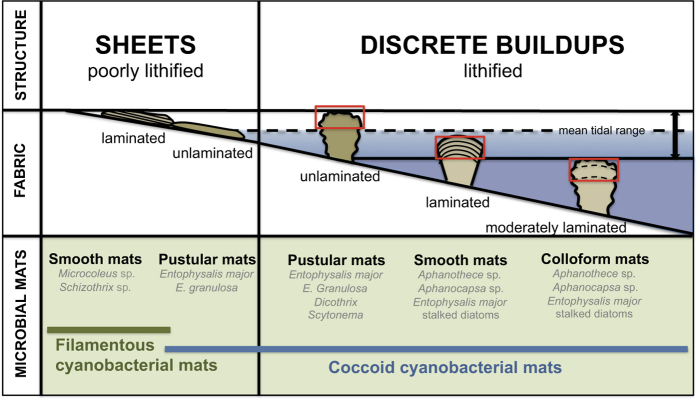
Revised growth model of microbial structures in Hamelin Pool. In contrast to previous studies, our model recognizes two types of pustular and smooth mats. Nearshore pustular and *filamentous* cyanobacterial smooth mats form unlaminated and laminated fabrics in poorly lithified sheets. Seaward pustular, smooth and colloform mats that are all enriched in *coccoid* cyanobacteria form unlaminated, well laminated and moderately laminated fabrics in discrete lithified buildups. The red boxes highlight surface fabrics formed by the designated mat types; multiple fabrics may be present in a single buildup. Note that in our model, well-laminated fabrics in discrete buildups are products of coccoid-dominated, rather than filamentous-dominated, smooth mats.

**Figure 4:**
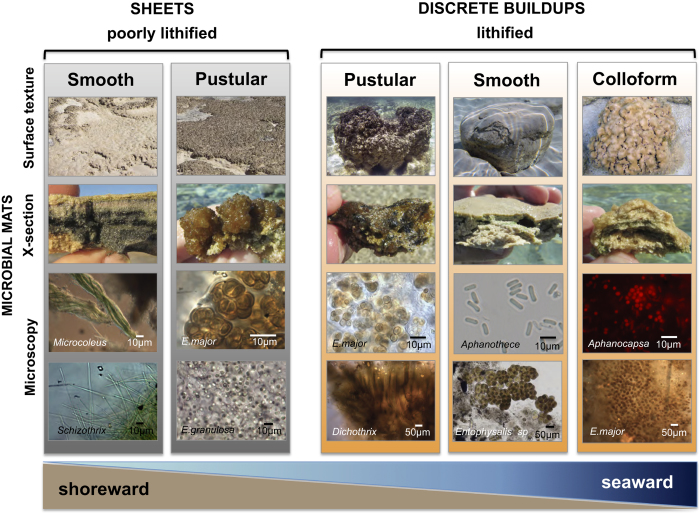
Micrographs of structure-forming microbial mats. Field photographs and photomicrographs illustrating surface textures, cross sectional views, and dominant microbes in the structure-forming microbial mats depicted in Fig. 3. Note that nearshore smooth mats forming laminated fabrics in poorly lithified sheets are dominated by filamentous cyanobacteria such as *Microcoleus* and *Schizothrix*, whereas seaward smooth mats forming laminated fabrics in discrete buildups are dominated by coccoid cyanobacteria, such as *Aphanothece* and *Entophysalis*. *Entophysalis*, typically associated primarily with pustular mats, is common in all mat types except the nearshore filamentous cyanobacterial mats.

As part of our multi-scale approach to studying stromatolite growth in Hamelin Pool, a two-pronged approach, including microscopy and molecular techniques, was used to document and characterize microbial mat communities. In a shoreward to seaward progression, we identified distinctive microbial communities associated with various mat types (Figs 3 and 4). Following historical usage^8^,^12^,^15^, we differentiated mats based on surface texture, recognizing pustular, smooth and colloform mat types. However, we also differentiated between mats forming poorly lithifying sheets versus those mats forming discrete lithified buildups.

#### Poorly Lithifying Sheets

Smooth and pustular mats forming poorly lithified sheets occupy the upper intertidal zone, exposed daily at low tide (Fig. 3). The smooth mats are well laminated and microscope analyses indicated that they are dominated by filamentous cyanobacteria, such as *Coleofasciculus* (i.e., *Microcoleus) chthonoplastes* and *Schizothrix helva* (Fig. 4). Pustular mats in the upper intertidal zone have unlaminated fabrics and are characterized by soft pustules of *Entophysalis major,* with clusters of *E. granulosa* (Fig. 4) and distinctive tetrads of smaller colonial coccoid cyanobacteria embedded in a thick matrix of exopolymeric substances (EPS).

#### Discrete lithified buildups

Progressing seaward, mats in the lower intertidal to subtidal zones with pustular, smooth and colloform surface textures lithify to form cemented fabrics that build discrete stromatolites (Fig. 3). Microscopic and molecular analyses revealed pronounced enrichments of coccoid cyanobacteria in all three of these stromatolite-building mat types. This finding contrasts with the traditional view^6^,^8^,^12^,^15^ that smooth mats forming well-laminated stromatolites are dominated by filamentous cyanobacteria.

**Figure 5:**
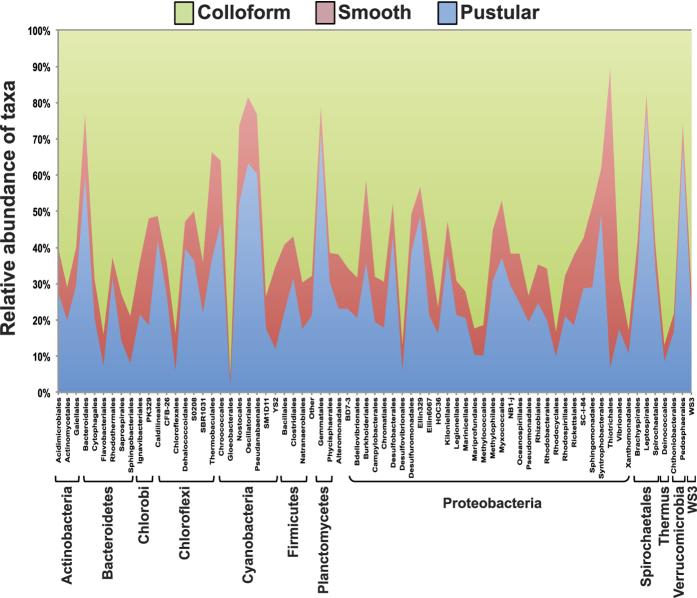
Taxonomic composition and diversity analyses of microbial mats associated with stromatolites. Relative abundance of dominant taxa that represent more than 0.1% of operational taxonomic units in all three distinct mat types: pustular^53^, smooth (red); and colloform (green) mat types.

Despite major similarities within the cyanobacterial populations of the three stromatolite-building mats, 16 S rRNA gene analysis revealed that, collectively, each mat type is a distinctive microbial community correlating to its water depth and geographic location. Amplicon libraries (n = 52) were generated for each mat type collected from four Stromatolite Provinces (Fig. 1; Supplementary Table S2–3). To calculate the species richness within the different samples rarefaction curves were generated for all 52 libraries and compared based on mat type (Supplementary Fig. S3a,b) and geographical location (Supplementary Fig. S3c,d). Results identified 800–1200 observed taxonomic units (OTUs; 97% identity) within the different mat types, which is higher than previous surveys of mats collected from Telegraph and Carbla Stations^14^,^16^,^17^. Differences were observed between the mat types at multiple taxonomic levels and the dominant taxa are visualized in Fig. 5; Supplementary Table S2; and Supplementary Figs S4, S5.

**Figure 6:**
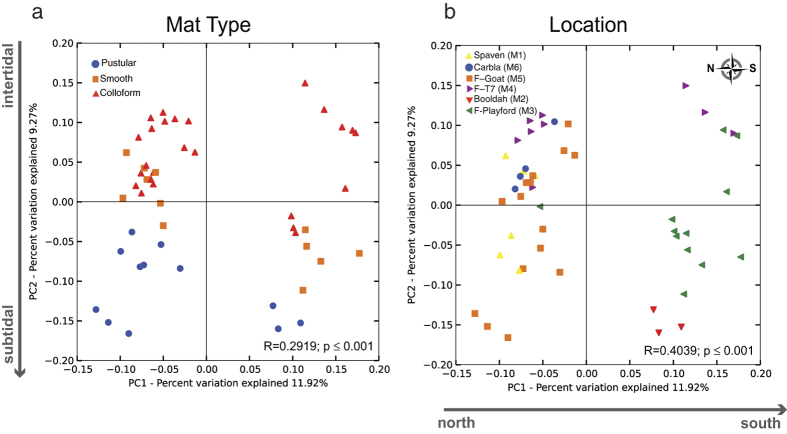
Principal coordinates analysis (PCoA) applied to the unweighted UniFrac distance matrices according to mat type and location. (**a**) The PCoA plot depicting differences in microbial diversity between the mat types and water depth. (**b**) The PCoA plot depicting differences in the microbial diversity and geographical location. Sample locations are shown in Supplementary Fig. S1.

To examine the impact of location on the different microbial mat types the 16 S rRNA gene libraries were analyzed with principal coordinates analysis generated from unweighted UniFrac distance matrices (Fig. 6a,b). The water depth of the mat type accounted for 11.92% of the total variation between communities (Fig. 6a; p ≤ 0.001; ANOSIM). The clustering pattern suggested a gradual transition of the bacterial diversity in those mats types located in the upper intertidal zone (i.e., pustular) compared to those that are located the lower intertidal to subtidal zone (i.e., smooth, colloform). In addition to water depth, geographical location had an impact on the microbial diversity of the communities (Fig. 6b; p ≤ 0.001; ANOSIM). There was a pronounced shift in the clustering pattern of mat samples collected from the southern provinces (i.e., Flagpole and Booldah) compared to those from the north (i.e., Spaven). These results suggest the local environment is a driver in mat diversity and may reflect changes in salinity, as salinity range increases from north to south in Hamelin Pool (Supplementary Table S1).

Microscope analysis of the pustular mats forming discrete heads show enrichments of coccoid *Entophysalis*-like cyanobacteria, specifically *E. major* and *E. granulosa*, coupled with filamentous cyanobacteria, *Scytonema* sp. and *Dichothrix* sp. (Fig. 5). Molecular analysis of the cyanobacterial diversity within the lithifying pustular mats showed the highest level of cyanobacterial diversity (p ≤ 0.01) of the different mat types (Supplementary Fig. S4).

The specific taxonomic hierarchies and relative abundance for all taxa within the lithifying smooth and colloform mats are represented as Krona plots in Supplementary Fig. S5. These lithifying smooth and colloform mats, which occur in the lower intertidal to subtidal zone, have a high relative abundance of coccoid cyanobacteria associated with the families Xenococcaceae and Pseudanabaenaceae (Fig. 5; Supplementary Fig. S5). In colloform mats, however, molecular data show an enrichment of coccoid bacteria associated with Gomphosphaeriaceae as well as recovered chloroplast sequences derived from phototrophic eukaryotes (e.g. diatoms; Supplementary Fig. S5). *Gomphosphera* sp. was, however, rarely observed by microscopy, which showed an abundance of *Aphanothece*, *Aphanocapsa* and *Entophysalis* (Fig. 4). It should be noted that *Entophysalis* and many other prominent genera are not in molecular databases, and hence were not identifiable in the molecular studies. *Entophysalis* species (*E. major, E. granulosa*) at Hamelin Pool can have different colored colonies as a result of light exposure (e.g., green, golden, black). In some cases, degraded forms *of E. major* may have been misidentified in previous studies as other species (e.g., *Gleocapsa punctata*, *Gloeothece vibrio*).

#### A revised growth model

Our revised model of mat zonation (Fig. 3) differs from classic models of previous studies in several ways. First, in contrast to former studies our model recognizes two types of ‘smooth mat’: (1) *filamentous* cyanobacterial smooth mats that form well laminated but poorly lithified sheets in upper intertidal zones; and (2) *coccoid* cyanobacterial smooth mats that form well laminated, lithified stromatolites in lower intertidal to shallow subtidal zones. It should be noted that many recent studies reporting on the composition of smooth mats^17^,^18^,^19^,^20^,^21^ sampled the nearshore intertidal *Microcoleus* (*filamentous*) mats, which form poorly lithified sheets but not lithified microbial buildups.

Additionally, descriptions of pustular mats are refined in the new model compared to previous studies. Rather than a single variety of pustular mat forming both sheets and discrete buildups^8^,^9^,^10^,^11^,^12^, our model distinguishes between poorly lithifying and lithifying pustular mat communities. Although both pustular mat communities are coccoid cyanobacterial mats dominated by *E. major*, the lithifying pustular mats are complex communities that also harbor EPS-rich filamentous cyanobacteria such as *Dichothrix* (Fig. 4), which is known to serve as loci for carbonate precipitation in other lithifying mat systems^22^. It is also noteworthy that *Entophysalis*, long recognized as the dominant component of pustular mats, is also prevalent in smooth and colloform coccoid mats. Indeed, *Entophysalis* is a key player in the Hamelin Pool microbial ecosystem.

The potential effects of seasonality, in particular variable temperature and salinity (Supplementary Table S1), on microbial mat composition in Hamelin Pool have yet to be rigorously evaluated. Although samples for microscopy and molecular analyses reported herein were primarily collected in Austral autumn (March and April, see Methods), field observations and limited microscopy of samples collected in Austral spring (November) showed no obvious changes in dominant cyanobacteria. Genetic and transcriptomic analyses should be conducted seasonably for a more complete understanding of microbial composition and function.

As in other modern environments, the stromatolite-forming microbial mat system in Hamelin Pool is dependent on environmental conditions that exclude macroalgae and other eukaryotic organisms that could overgrow and outcompete the prokaryotic stromatolite-forming microbial mats. In Hamelin Pool, these conditions are provided by hypersalinity, together with harsh conditions associated with large fluctuations in salinity, temperature and exposure. Some eukaryotes such as diatoms and *Acetabularia*, which are able to withstand these harsh conditions, are common but the role of these eukaryotes in accretion of Shark Bay stromatolites is presently unknown. In Bahamian stromatolites, built by filamentous cyanobacterial mats, diatoms and eukaryotes are not essential for accretion^23^,^24^.

### Pervasive precipitation of microbial micrite

**Figure 7:**
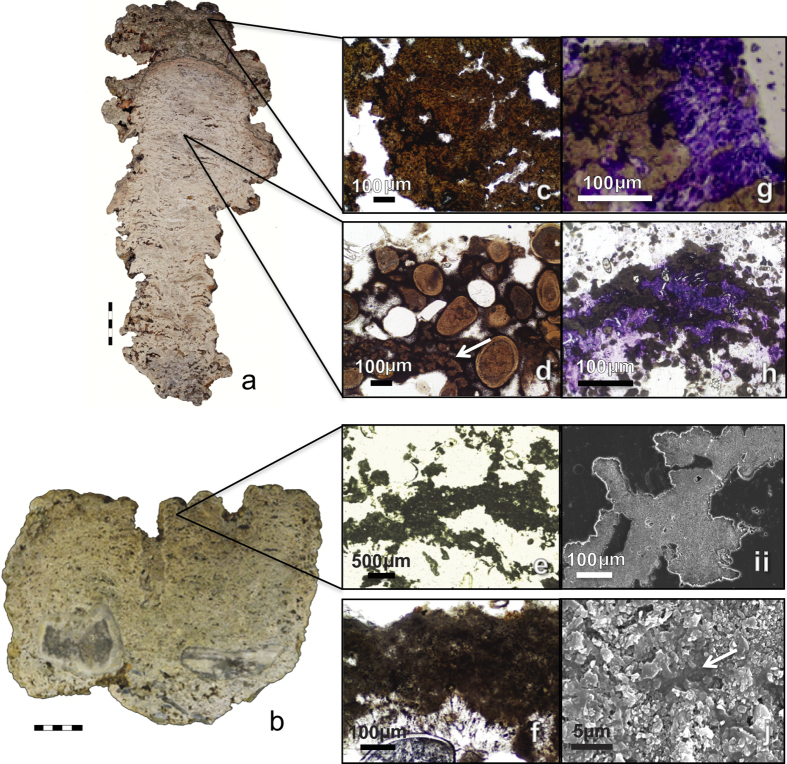
Microbial micrite in Hamelin Pool stromatolites. (**a,b**) Polished slabs showing vertical section of a stromatolite with a well laminated core and an upper green, unlaminated cap (**a**) and a moderately well laminated stromatolite (**b**). (**c–f**) Thin section photomicrographs depicting massive red brown micrite (**c**), the dominant component of the cap in (**a**); red brown micrite cement (**d**, arrow), forming cemented grain layers in the core of **(a**); grey peloidal micrite (**e,f**) forming a laminated framework in (**b**). (**g,h**) Thin sections stained with methylene blue to highlight organics (purple); note intimate relationship between micrite and coccoid cyanobacteria, dominantly *Entophysalis*. (**i,j**) Scanning electron photomicrographs of grey peloidal micritic; note the lack of microborings in (**i**) and the EPS-rich matrix (arrow) enveloping micrite crystals in (**j**).

Field observations together with laboratory analyses of microbial mats and internal fabrics of 45 stromatolite heads collected from Hamelin Pool indicate that pervasive precipitation of micrite within microbial mats forms stromatolite framework and cements (Fig. 7). These results are contrary to the traditional view that Hamelin Pool stromatolites are accreted primarily by trapping and binding of carbonate sand^8^,^11^,^25^,^26^,^27^. Although microbial micrite is mentioned in previous studies^6^,^7^,^8^,^11^, only one report^28^ has documented Hamelin stromatolites with laminated micritic framework. In contrast, observations in the present study offer extensive evidence that microbial precipitation is pervasive and of fundamental importance in stromatolite accretion in Hamelin Pool.

Detailed microscopy analyses revealed two varieties of precipitated miocrobial micrite forming primary framework and cement in Hamelin Pool stromatolites: a red brown micrite with dark inclusions and a grey peloidal micrite (Fig. 7); red and grey are colors as observed in plane polarized light. X-ray diffraction indicated that both micrites are aragonite. In petrographic thin sections, precipitated peloidal micrite was often difficult to differentiate from altered skeletal grains and detrital peloids. Scanning electron microscopy (SEM) revealed that most altered grains were heavily microbored, whereas precipitated micritic was at least initially, not microbored (Fig. 7).

Primary depositional fabrics of all stromatolite heads collected during this study are composed of variable portions of sediment plus one or both types of microbial micrite (i.e. red brown or grey peloidal micrite)^29^. Moreover, this micrite comprises an estimated 20–50% of most stromatolites and >80% of some heads^29^.

Red brown micrite forms massive knobby protrusions that cap some stromatolites colonized by pustular mat in the upper intertidal zone (Fig. 7a,c). This dense micrite likely represents calcified *Entophysalis* mat (Fig. 7g,h, Supplmental Fig. S6) and the dark inclusions may be shriveled entombed cells^27^,^28^,^29^,^30^. Red brown micrite also forms clots and laminae within stromatolites, in some cases cementing accreted sediment grains (Fig. 7d). The common occurrence of red brown micrite within the stromatolites is consistent with morphological observations of the relative high abundance of the coccoid *Entophysalis* spp. in the lithifying, stromatolite-forming mats in Hamelin Pool (Fig. 4).

Grey peloiodal micrite forms laminae and clots in gelatinous varieties of smooth and colloform mats in lower intertidal to subtidal zones. These mats form on stromatolite heads and cover vast areas of undulating low relief pavement. In some stromatolites, precipitated laminae of grey peloidal micrite comprise over 80% of the structure, forming a micritic framework (Fig. 7b,e,f); in other heads, the precipitates form short laminae within crusty rinds several centimeters thick that coat stromatolites. Grey peloidal micrite also cements accreted sediment within the stromatolites.

Preferential precipitation of micrite in specific mat types is evidence of microbial influence. However, the exact mechanisms that triggered the carbonate precipitation are not constrained. Carbonate precipitation could be linked to heterotrophic activity (i.e., sulfate reduction) or photosynthetic CO_2_ uptake, both of which can increase the local carbonate saturation state. Release of EPS-bound calcium during remineralization of organic matter in microbial mats can also result in carbonate precipitation^31^. It is, however, also important to stress that Hamelin Pool is primed for carbonate precipitation. Foremost, Hamelin Pool is a warm shallow carbonate basin in which degassing might occur. Additionally, groundwater injection may trigger carbonate precipitation. Groundwater with an alkalinity of up to 5.9 meq. l^−1^ has been collected from Booldah Well^32^ and influx of this highly alkaline groundwater into Hamelin Pool, combined with high water temperatures in Austral summer months, may enhance precipitation of micrite. Episodic precipitation, whether microbial and/or environmentally controlled, may result in lamination (cf., ref. 23).

### Window into the Precambrian

**Figure 8:**
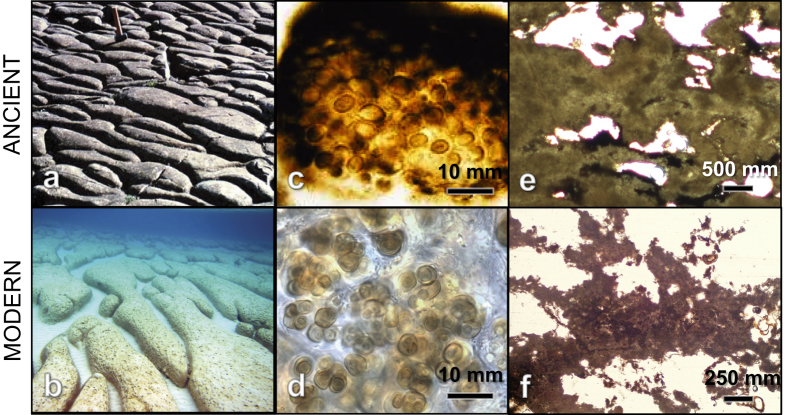
Images comparing stromatolite morphology, microbes, and microfabrics of Hamelin Pool (modern) and Precambrian stromatolites (ancient). (**a**) 1.9 billion year old stromatolites at Great Slave Lake, Northwest Territories; from Hoffman 1967,32 reprinted with permission from American Association for the Advancement of Science. (**b**) Elongate nested structures in Spaven Province. (**c**) *Eoentophysalis* in stromatolitic chert from the 2.1 billion year old Kasegalik Formation at Belcher Islands, Canada; photo credit H. Hoffman, reprinted with permission from Precambrian Paleobiology Research Group. (**d**) *Entophysalis major* from Hamelin Pool. (**e**) Neoproterozoic stromatolites from the Amadeus Basin, Western Australia exhibiting clotted peloidal structure. Photo credit K. Grey, H.J. Allen, image courtesy of the Geological Survey of Western Australia, Department of Mines and Petroleum, Western Australia^©^, State of Western Australia717 2015. (**f**) lamina of clotted peloidal micrite in Hamelin stromatolite; see also Fig. 7f.

New insights regarding stromatolite growth in Hamelin Pool present opportunities for comparative sedimentological research advancing understanding of early Earth. The diversity of morphologies in the eight Stromatolite Provinces (Figs 1 and 2 and Supplementary Fig. S2) provides a unique opportunity for investigating environmental and/or biological processes determining stromatolite morphology. Extending pioneer work by Logan, Playford, Hoffman and others^33^, integrated studies of morphometric, environmental, and biological data in the eight provinces could lead to improved understanding of stromatolite morphogenesis. Of particular note, previously unreported, elongate nested subtidal structures of Spaven Province are remarkably similar to 1.9 billion year old longitudinal stromatolites at Great Slave Lake, Northwest Territories^34^ (Fig. 8a,b).

Another attribute of Hamelin Pool stromatolites that has implications for studies of ancient structures is recognition that lithified, well laminated buildups in Hamelin are products of coccoid cyanobacterial mats, with filamentous cyanobacterial mats forming poorly lithified sheets. Moreover, *Entophysalis,* a coccoid that is common in smooth, colloform and pustular lithifying mats (Figs 3 and 4) has an ancient lineage: early and middle Proterozoic stromatolite assemblages were dominated by *Eoentophysalis* sp., which is a precursor to modern *Entophysalis*^35^ (Fig. 8c,d).

Finally, pervasive precipitation of microbial micrite forming stromatolite framework and cements in Hamelin Pool has an intriguing relevance for Precambrian environments. Because of their coarse grained nature, Hamelin Pool stromatolites have traditionally been considered inappropriate analogs for Precambrian stromatolites, which are typically micritic^26^. Recognition that precipitation of microbial micrite is an important mechanism of stromatolite accretion in Hamelin Pool, at times forming laminated micritic framework (Fig. 8), challenges this claim. With millimeter-thick laminae, this framework is coarser than the sub-millimeter lamination typical of many Precambrian stromatolites. Nevertheless, internal fabrics of Neoproterozoic stromatolites from Western Australia, for example, show a strong resemblance to the clotted peloidal textures of grey micrite in Hamelin stromatolites (Figs 7f and 8e,f).

In summary, at all scales, Hamelin Pool is a spectacular living laboratory. In terms of morphological diversity, microbial communities, and micrite precipitation, Hamelin stromatolites offer a potential window into Precambrian environments, providing a basis for reconstructing ancient environments and understanding how benthic microbial communities interacted with these environments. Our initial work illustrated that even basic aspects of this landmark geobiological system are poorly known. Thus, this unique ecosystem should be studied intensively before it is altered by sea level rise in the coming decades.

## Methods

### Field work, sample collection, and environmental data

Fieldwork using small boats was conducted during three, two-month field seasons (March and April 2012–2014), with additional site visits in November 2013 and 2014. A real time kinematic (RTK) survey along set transects was conducted using a Trimble R6 base station paired with an R8 rover to establish absolute elevations. Extensive in-water observations, supported by underwater photography, spot dives and manta tows, established high density ground truth coverage (Supplementary Fig. S1). Sample collection included 45 stromatolite heads, 84 microbial mat sections, 35 subtidal pavements, and 250 sediment samples.

Single beam sonar soundings were collected using a high precision survey grade single beam echo sounder (Ohmex SonarMite v3 EchoSounder - Legacy) coupled directly to a Trimble rover. The rover was hard mounted on a boat at a fixed height above the water surface with the transducer mounted directly to the rover pole. Depths were subtracted from the measured water surface elevations giving an absolute elevation at each sample point. The nominal zero elevation was established using RTK survey and post processed correlate with the Hamelin Pool benchmark north of Flagpole Landing (A906; 4.253 m below Australian Height Datum). The Trimble R10 GNSS receiver has a built in tilt sensor that enabled real-time tracking of the rover pole tilt to help eliminate errors resulting from excessive tilt due to wind driven swells. Water surface elevations were checked at a random sampling throughout bathymetric data collection to exclude data outliers.

To construct the bathymetry map, sonar data were processed in combination with Landsat 8 imagery using ENVI’s SPEAR Relative Water Depth tool, which employs the Stumpf and Holderied^36^ bottom albedo-independent bathymetry algorithm. The rendering was calibrated to absolute depth using the Log Ratio Transform and an imported ASCII file of absolute elevations collected from the single beam sonar survey.

To log environmental parameters, star-pickets were installed in five key locations around the Hamelin Pool (Fig. 1) to record temperature, salinity and pressure (i.e. tidal data). Loggers were attached to the pickets at ~2 m depth and set to record at 30 min intervals for 18 months (March 2013 to November 2014). Salinity data were collected using AquiStar CT2x submersible smart sensors capable of collecting a wide range of conductivity measurements (10,000 to 100,000 μSiemans/cm). Temperature data were collected using Onset HOBO TidbiT v2 Water Temperature Data Loggers (UTBI-001). Tidal data were collected using Solinst Model 3001 Levelogger Junior Edge and calibrated against local Hamelin Pool barometric pressure.

### Microscopy

Samples were collected and maintained in seawater for immediate microscope analysis, with a subset preserved on site with 2.5% glutaraldehyde or 4% formalin in filtered seawater. Preserved samples were kept chilled and in the dark. Light micrographs were taken on an Olympus BX51 fluorescence microscope with a Micropublisher Camera (Q Imaging, Surry BC)^37^.

### Molecular analyses of microbial communities using barcoded 16 S rRNA gene library

Cores (8 mm × 8 mm) of the microbial mat samples were collected in triplicate using a Harris Uni-corer (Ted Pella, Redding, CA), placed in RNAlater (Life Technologies, Carlsbad, CA), and stored at −80 °C until processed. DNA was extracted from each core in triplicate using a modified xanthogenate method as previously described^38^. For each sample, PCR reactions were conducted in triplicate using conditions as previously described^39^ and pooled in equimolar concentrations. The primer for each reaction included a unique barcode (Supplementary Table S3) that targeted the V1-2 region in bacteria. Three replicate amplicon libraries were generated for each microbial mat sample and sequenced using the Illumina GAIIx platform producing a total of 2,262,282 high-quality filtered sequences, which were deposited in the NCBI sequencing read archive under project number SRP055055.

### Bioinformatics and statistical analyses

The recovered 16 S rRNA gene barcoded amplicon sequences were analyzed using QIIME v.1.8.0^40^. Sequences were quality filtered and demultiplexed using the QIIME script *split_library.fastq.py* using suitable parameters for Illumina reads, as previously described^41^. The parameters included: minimum number of consecutive high-quality base calls to include a read (per single end read) as a fraction of the input read length was 0.75; minimum Phred quality score of 19; maximum number of consecutive low quality base calls allowed before truncating a read was 3; maximum number of errors in barcode was 1.5. The filtered reads were assigned to operational taxonomic units (OTUs) using an open-reference OTU picking approach based on sequence similarity using UCLUST against the Greengenes database (v. 13_8)^42^ at 97% identity. A representative set of sequences was taken for each OTU and a taxonomic classification was performed with the Ribosomal Database Project (RDP) classifier 2.2^43^. The representative sequences were then aligned using PyNAST^44^ to the Greengenes Core reference alignment and a phylogenetic tree was built using FastTree^45^. The generated OTU table was used for taxonomic comparison, filtering the OTU at 0.1% and producing taxonomic pie charts using the Krona tool^46^. The OTU table and the phylogenetic tree were used as input for downstream phylogenetic community analyses. Community diversity analyses were performed at a rarefaction depth of 4,852 sequences per sample. The taxonomic OTU table was rarefied at 4,852 sequences/sample at 10 levels, each repeated for 1,000 iterations. Alpha diversity indices were computed on every rarefied OTU tables using observed species and Faith’s phylogenetic diversity (PD) metric^47^, and the average result was used to build rarefaction curves. Beta diversity comparisons were computed as Principal Coordinates Analyses (PCoA) generated from unweighted UniFrac distance matrices^48^. Analysis of Similarity Statistics (ANOSIM)^49^ and non-parametric multivariate ANOVA (ADONIS) using UniFrac distance matrices were used to test the significance of differences between the different samples. The alpha diversity significance was tested with a non-parametric two-sample t-test using 999 Monte Carlo permutations to calculate the p-values. To compare the OTU frequencies among sample groups, a Kruskal-Wallis one-way analysis of variance test^50^ was performed and the generated p-values were corrected using Bonferroni method^51^

### Microfabrics

Approximately 300 2 × 3 inch petrographic thin sections were made from 45 stromatolite heads. Additionally, thin sections were made from ~250 sediment samples, and 50 mat samples. Thin sections were analyzed using a petrographic microscope (Olympus BH-2) with plane-polarized and cross-polarized transmitted light.

Scanning electron microscopy was used to further characterize thin sections. Sections were etched in 1% HCl for 10–15 seconds before being coated with palladium and analyzed using a Philips XL30 ESEM-FEG. Carbonate mineralogy was determined using X-ray diffraction following Swart and Melim^52^. Samples were homogenized using a mortar and pestle then smear mounted onto glass slides which were then scanned between 23 and 32° 2θ with CuKα radiation using a Panalytical X-Pert Pro.

## Additional Information

**How to cite this article**: Suosaari, E. P. *et al.* New multi-scale perspectives on the stromatolites of Shark Bay, Western Australia. *Sci. Rep.* **6**, 20557; doi: 10.1038/srep20557 (2016).

## Supplementary Material

Supplementary Information
