# Supplementary material for: New multi-scale perspectives on the stromatolites of Shark Bay, Western Australia
Source: Sci Rep. 2016 Feb 3;6:20557. doi: 10.1038/srep20557 (PMC4738353; doi:10.1038/srep20557)
Supplement: Supplementary Information [file srep20557-s1.pdf]

Supplemental Material

New multi-scale perspectives on the stromatolites of Shark Bay, Western Australia

Running Title: New insights into Shark Bay stromatolites

E.P. Suosaari[1], R.P. Reid[1*], P.E. Playford[2], J.S. Foster[3], J.F. Stolz[4], G. Casaburi[3], P.D. Hagan[1], V. Chirayath[5], I.G. Macintyre[6], N.J. Planavsky[7], and G.P. Eberli[1]

[1]Rosenstiel School of Marine and Atmospheric Science, University of Miami, Miami, Florida 33158, USA

[2]Geological Survey of Western Australia, Perth, Western Australia

[3]Department of Microbiology and Cell Science, University of Florida, Space Life Science Lab, Merritt Island, Florida, 32953, USA

[4]Department of Biological Sciences, Duquesne University, Pittsburgh, Pennsylvania, 15282, USA

[5]NASA Ames Research Center, Moffett Field, CA, 94035, USA

[6]National Museum of Natural History, Smithsonian Institution, Washington DC 20013, USA

[7]Department of Geology and Geophysics, Yale University, New Haven, CT, 06520, USA

*Corresponding Author

Email: preid@rsmas.miami.edu

Supplementary Table S1. Environmental parameters of salinity, temperature and pressure (i.e. tidal data) as recorded for 18 months (March 2013 to November 2014) by loggers deployed at 2 m depth in five key locations (see Fig. 1). Current meters collected data at 4-minute intervals over a 6-week period in March-April 2013.

		NW	W	S	E	NE
**Sal (‰)**	Min	29.2	27.2	45.3	15.8	32.4
	Max	75.8	78.3	88.1	81.9	80.8
	Range	46.6	51.1	42.8	66.1	48.4
	Average	63.0	67.3	69.4	65.6	65.6
**Temp (°C)**	Min	14.2	14.6	11.2	14.3	13.9
	Max	31.5	29.9	33.0	32.0	32.0
	Range	17.3	15.3	21.8	17.7	18.1
	Average	22.7	22.2	22.6	23.0	23.2
**Tide (m)**	Min	-0.79	-0.58	failed logger	-0.60	-0.80
	Max	0.86	1.29		1.44	1.23
	Range	1.65	1.87		2.04	2.03
	Average	-0.04	0.28		0.26	0.04
**Current (m/s)**	Min	0.00	0.00	0.00	0.00	0.00
	Max	0.63	0.73	0.57	0.60	0.74
	Average	0.15	0.13	0.07	0.12	0.14

Supplementary Table S2. Statistical comparison at phylum and family level between mat types. The p-values were generated using a Kruskal-Wallis test with a Bonferroni correction and ranged from 0.01 - 0.05 (*), 0.001 - 0.01 (**) and <0.001 (***).

Phylum:	Smooth / Colloform	p-values ranges	Smooth / Pustular	p-values ranges	Pustular / Colloform	p-values ranges
Actinobacteria	1		1		1	
Bacteroidetes	1		0.718595717		0.007257473	**
Chloroflexi	1		1		1	
Cyanobacteria	0.084549081		1		0.005736052	**
Firmicutes	1		1		1	
GN04	0.254002645		1		0.254002645	
Planctomycetes	1		0.007571473	**	1	
Proteobacteria	1		1		1	
Verrucomicrobia	1		1		1	

Family:	Smooth / Colloform	p-values ranges	Smooth / Pustular	p-values ranges	Pustular / Colloform	p-values ranges
A4b	1		1		1	
Anaerobrancaceae	1		1		1	
Crenotrichaceae	1		1		1	
Cytophagaceae	0.14833202		1		0.075175572	
Dehalococcoidaceae	1		0.370015239		0.041655194	*
Erythrobacteraceae	0.349639043		1		0.004747111	**
Flammeovirgaceae	1		1		1	
Flavobacteriaceae	1		1		0.02892363	*
Gomphosphaeriaceae	1		1		1	
Hyphomicrobiaceae	1		1		1	
Hyphomonadaceae	1		1		1	
Marinicellaceae	1		1		1	
Nannocystaceae	1		1		1	
Nostocaceae	1		1		0.002074975	**
OM27	1		1		1	
Phormidiaceae	0.059518928	*	0.133642445		0.000564701	***
Piscirickettsiaceae	0.041655194	*	1		1	
Pseudanabaenaceae	1		1		0.019925043	*
Rhodobacteraceae	1		1		1	
Rhodospirillaceae	1		1		1	
Rhodothermaceae	1		0.248529503		1	
Saprospiraceae	1		1		0.206045192	
SJA/101	1		1		0.165616246	
Thermobaculaceae	1		1		0.06678011	
Xanthomonadaceae	1		1		1	
Xenococcaceae	1		1		0.283650175	

Supplementary Table S3. Primers used for generating bacterial 16S rRNA barcoded library, associated mat type and location.

Primer Name	Illumina adapter (GAIIx)	Linker	Barcode	Illumina Sequencing Primer	16S rRNA gene primer sequence*	Mat Type	Map ID**	Location
Bac338R	AATGATACGGCGACCACCGA	GATCT		ACACTCTTTCCCTACACGACGCTCTTCCGATCT	TGCTGCCTCCCGTAGGAGT	all samples		all locations
Bac27F-16	CAAGCAGAAGACGGCATACGA	GAT	TTATATG	GTGACTGGAGTTCAGACGTGTGCTCTTCCGATCT	AGAGTTTGATCCTGGCTCAG	Pustular	M1	Spaven
Bac27F-17	CAAGCAGAAGACGGCATACGA	GAT	GTTAATG	GTGACTGGAGTTCAGACGTGTGCTCTTCCGATCT	AGAGTTTGATCCTGGCTCAG	Pustular	M1	Spaven
Bac27F-18	CAAGCAGAAGACGGCATACGA	GAT	AACAATG	GTGACTGGAGTTCAGACGTGTGCTCTTCCGATCT	AGAGTTTGATCCTGGCTCAG	Pustular	M1	Spaven
Bac27F-19	CAAGCAGAAGACGGCATACGA	GAT	CAGCATG	GTGACTGGAGTTCAGACGTGTGCTCTTCCGATCT	AGAGTTTGATCCTGGCTCAG	Smooth	M1	Spaven
Bac27F-20	CAAGCAGAAGACGGCATACGA	GAT	TGTGCTG	GTGACTGGAGTTCAGACGTGTGCTCTTCCGATCT	AGAGTTTGATCCTGGCTCAG	Smooth	M1	Spaven
Bac27F-21	CAAGCAGAAGACGGCATACGA	GAT	GCCGCTG	GTGACTGGAGTTCAGACGTGTGCTCTTCCGATCT	AGAGTTTGATCCTGGCTCAG	Smooth	M1	Spaven
Bac27F-22	CAAGCAGAAGACGGCATACGA	GAT	AATTCTG	GTGACTGGAGTTCAGACGTGTGCTCTTCCGATCT	AGAGTTTGATCCTGGCTCAG	Colloform	M6	Carbla
Bac27F-23	CAAGCAGAAGACGGCATACGA	GAT	CCATCTG	GTGACTGGAGTTCAGACGTGTGCTCTTCCGATCT	AGAGTTTGATCCTGGCTCAG	Colloform	M6	Carbla
Bac27F-24	CAAGCAGAAGACGGCATACGA	GAT	CTAGGAG	GTGACTGGAGTTCAGACGTGTGCTCTTCCGATCT	AGAGTTTGATCCTGGCTCAG	Colloform	M6	Carbla
Bac27F-25	CAAGCAGAAGACGGCATACGA	GAT	CCTTGAG	GTGACTGGAGTTCAGACGTGTGCTCTTCCGATCT	AGAGTTTGATCCTGGCTCAG	Colloform	M6	Carbla
Bac27F-26	CAAGCAGAAGACGGCATACGA	GAT	GGAAGAG	GTGACTGGAGTTCAGACGTGTGCTCTTCCGATCT	AGAGTTTGATCCTGGCTCAG	Colloform	M6	Carbla
Bac27F-27	CAAGCAGAAGACGGCATACGA	GAT	CACAGAG	GTGACTGGAGTTCAGACGTGTGCTCTTCCGATCT	AGAGTTTGATCCTGGCTCAG	Colloform	M6	Carbla
Bac27F-28	CAAGCAGAAGACGGCATACGA	GAT	TGGCGAG	GTGACTGGAGTTCAGACGTGTGCTCTTCCGATCT	AGAGTTTGATCCTGGCTCAG	Colloform	M4	Flagpole (T7)
Bac27F-29	CAAGCAGAAGACGGCATACGA	GAT	GATCGAG	GTGACTGGAGTTCAGACGTGTGCTCTTCCGATCT	AGAGTTTGATCCTGGCTCAG	Colloform	M4	Flagpole (T7)
Bac27F-30	CAAGCAGAAGACGGCATACGA	GAT	TATGTAG	GTGACTGGAGTTCAGACGTGTGCTCTTCCGATCT	AGAGTTTGATCCTGGCTCAG	Colloform	M4	Flagpole (T7)
Bac27F-37	CAAGCAGAAGACGGCATACGA	GAT	CTCCAAG	GTGACTGGAGTTCAGACGTGTGCTCTTCCGATCT	AGAGTTTGATCCTGGCTCAG	Colloform	M4	Flagpole (T7)
Bac27F-38	CAAGCAGAAGACGGCATACGA	GAT	ATTGCAG	GTGACTGGAGTTCAGACGTGTGCTCTTCCGATCT	AGAGTTTGATCCTGGCTCAG	Colloform	M4	Flagpole (T7)
Bac27F-39	CAAGCAGAAGACGGCATACGA	GAT	TAATCAG	GTGACTGGAGTTCAGACGTGTGCTCTTCCGATCT	AGAGTTTGATCCTGGCTCAG	Colloform	M4	Flagpole (T7)
Bac27F-40	CAAGCAGAAGACGGCATACGA	GAT	AGCTCAG	GTGACTGGAGTTCAGACGTGTGCTCTTCCGATCT	AGAGTTTGATCCTGGCTCAG	Pustular	M5	Flagpole (Goat)
Bac27F-41	CAAGCAGAAGACGGCATACGA	GAT	GCTACAG	GTGACTGGAGTTCAGACGTGTGCTCTTCCGATCT	AGAGTTTGATCCTGGCTCAG	Pustular	M5	Flagpole (Goat)
Bac27F-42	CAAGCAGAAGACGGCATACGA	GAT	AAGCCAG	GTGACTGGAGTTCAGACGTGTGCTCTTCCGATCT	AGAGTTTGATCCTGGCTCAG	Pustular	M5	Flagpole (Goat)
Bac27F-43	CAAGCAGAAGACGGCATACGA	GAT	TCTGGCG	GTGACTGGAGTTCAGACGTGTGCTCTTCCGATCT	AGAGTTTGATCCTGGCTCAG	Pustular	M5	Flagpole (Goat)
Bac27F-44	CAAGCAGAAGACGGCATACGA	GAT	GTCGGCG	GTGACTGGAGTTCAGACGTGTGCTCTTCCGATCT	AGAGTTTGATCCTGGCTCAG	Pustular	M5	Flagpole (Goat)
Bac27F-45	CAAGCAGAAGACGGCATACGA	GAT	AAGAGCG	GTGACTGGAGTTCAGACGTGTGCTCTTCCGATCT	AGAGTTTGATCCTGGCTCAG	Pustular	M5	Flagpole (Goat)
Bac27F-46	CAAGCAGAAGACGGCATACGA	GAT	CTTAGCG	GTGACTGGAGTTCAGACGTGTGCTCTTCCGATCT	AGAGTTTGATCCTGGCTCAG	Smooth	M5	Flagpole (Goat)
Bac27F-47	CAAGCAGAAGACGGCATACGA	GAT	CCGCGCG	GTGACTGGAGTTCAGACGTGTGCTCTTCCGATCT	AGAGTTTGATCCTGGCTCAG	Smooth	M5	Flagpole (Goat)
Bac27F-48	CAAGCAGAAGACGGCATACGA	GAT	TAACGCG	GTGACTGGAGTTCAGACGTGTGCTCTTCCGATCT	AGAGTTTGATCCTGGCTCAG	Smooth	M5	Flagpole (Goat)
Bac27F-49	CAAGCAGAAGACGGCATACGA	GAT	CCAGTCG	GTGACTGGAGTTCAGACGTGTGCTCTTCCGATCT	AGAGTTTGATCCTGGCTCAG	Colloform	M5	Flagpole (Goat)
Bac27F-50	CAAGCAGAAGACGGCATACGA	GAT	TGGTTCG	GTGACTGGAGTTCAGACGTGTGCTCTTCCGATCT	AGAGTTTGATCCTGGCTCAG	Colloform	M5	Flagpole (Goat)
Bac27F-51	CAAGCAGAAGACGGCATACGA	GAT	AGAATCG	GTGACTGGAGTTCAGACGTGTGCTCTTCCGATCT	AGAGTTTGATCCTGGCTCAG	Colloform	M5	Flagpole (Goat)
Bac27F-55	CAAGCAGAAGACGGCATACGA	GAT	GCATACG	GTGACTGGAGTTCAGACGTGTGCTCTTCCGATCT	AGAGTTTGATCCTGGCTCAG	Colloform	M5	Flagpole (Goat)
Bac27F-56	CAAGCAGAAGACGGCATACGA	GAT	GGCAACG	GTGACTGGAGTTCAGACGTGTGCTCTTCCGATCT	AGAGTTTGATCCTGGCTCAG	Colloform	M5	Flagpole (Goat)
Bac27F-57	CAAGCAGAAGACGGCATACGA	GAT	GATGCCG	GTGACTGGAGTTCAGACGTGTGCTCTTCCGATCT	AGAGTTTGATCCTGGCTCAG	Colloform	M5	Flagpole (Goat)
Bac27F-58	CAAGCAGAAGACGGCATACGA	GAT	TCGACCG	GTGACTGGAGTTCAGACGTGTGCTCTTCCGATCT	AGAGTTTGATCCTGGCTCAG	Smooth	M3	Flagpole (Playford)
Bac27F-59	CAAGCAGAAGACGGCATACGA	GAT	ACGTGGT	GTGACTGGAGTTCAGACGTGTGCTCTTCCGATCT	AGAGTTTGATCCTGGCTCAG	Smooth	M3	Flagpole (Playford)
Bac27F-60	CAAGCAGAAGACGGCATACGA	GAT	CTATGGT	GTGACTGGAGTTCAGACGTGTGCTCTTCCGATCT	AGAGTTTGATCCTGGCTCAG	Smooth	M3	Flagpole (Playford)
Bac27F-61	CAAGCAGAAGACGGCATACGA	GAT	CGTAGGT	GTGACTGGAGTTCAGACGTGTGCTCTTCCGATCT	AGAGTTTGATCCTGGCTCAG	Smooth	M3	Flagpole (Playford)
Bac27F-62	CAAGCAGAAGACGGCATACGA	GAT	GACAGGT	GTGACTGGAGTTCAGACGTGTGCTCTTCCGATCT	AGAGTTTGATCCTGGCTCAG	Smooth	M3	Flagpole (Playford)
Bac27F-63	CAAGCAGAAGACGGCATACGA	GAT	AGACGGT	GTGACTGGAGTTCAGACGTGTGCTCTTCCGATCT	AGAGTTTGATCCTGGCTCAG	Smooth	M3	Flagpole (Playford)
Bac27F-67	CAAGCAGAAGACGGCATACGA	GAT	TAGTTGT	GTGACTGGAGTTCAGACGTGTGCTCTTCCGATCT	AGAGTTTGATCCTGGCTCAG	Colloform	M3	Flagpole (Playford)
Bac27F-68	CAAGCAGAAGACGGCATACGA	GAT	AGCTTGT	GTGACTGGAGTTCAGACGTGTGCTCTTCCGATCT	AGAGTTTGATCCTGGCTCAG	Colloform	M3	Flagpole (Playford)
Bac27F-69	CAAGCAGAAGACGGCATACGA	GAT	CCGATGT	GTGACTGGAGTTCAGACGTGTGCTCTTCCGATCT	AGAGTTTGATCCTGGCTCAG	Colloform	M3	Flagpole (Playford)
Bac27F-73	CAAGCAGAAGACGGCATACGA	GAT	CACTAGT	GTGACTGGAGTTCAGACGTGTGCTCTTCCGATCT	AGAGTTTGATCCTGGCTCAG	Colloform	M4	Flagpole (T7)
Bac27F-74	CAAGCAGAAGACGGCATACGA	GAT	GTGAAGT	GTGACTGGAGTTCAGACGTGTGCTCTTCCGATCT	AGAGTTTGATCCTGGCTCAG	Colloform	M4	Flagpole (T7)
Bac27F-75	CAAGCAGAAGACGGCATACGA	GAT	TCCAAGT	GTGACTGGAGTTCAGACGTGTGCTCTTCCGATCT	AGAGTTTGATCCTGGCTCAG	Colloform	M4	Flagpole (T7)
Bac27F-76	CAAGCAGAAGACGGCATACGA	GAT	CGGCAGT	GTGACTGGAGTTCAGACGTGTGCTCTTCCGATCT	AGAGTTTGATCCTGGCTCAG	Colloform	M3	Flagpole (Playford)
Bac27F-77	CAAGCAGAAGACGGCATACGA	GAT	GTTGCGT	GTGACTGGAGTTCAGACGTGTGCTCTTCCGATCT	AGAGTTTGATCCTGGCTCAG	Colloform	M3	Flagpole (Playford)
Bac27F-78	CAAGCAGAAGACGGCATACGA	GAT	CGAGCGT	GTGACTGGAGTTCAGACGTGTGCTCTTCCGATCT	AGAGTTTGATCCTGGCTCAG	Colloform	M3	Flagpole (Playford)
Bac27F-79	CAAGCAGAAGACGGCATACGA	GAT	CCTTCGT	GTGACTGGAGTTCAGACGTGTGCTCTTCCGATCT	AGAGTTTGATCCTGGCTCAG	Pustular	M2	Booldah
Bac27F-80	CAAGCAGAAGACGGCATACGA	GAT	TGGACGT	GTGACTGGAGTTCAGACGTGTGCTCTTCCGATCT	AGAGTTTGATCCTGGCTCAG	Pustular	M2	Booldah
Bac27F-81	CAAGCAGAAGACGGCATACGA	GAT	GAGCCGT	GTGACTGGAGTTCAGACGTGTGCTCTTCCGATCT	AGAGTTTGATCCTGGCTCAG	Pustular	M2	Booldah

*Bacterial 16S rRNA gene primer sequences derived from Suzuki et al., 1996

** Map ID corresponds to location on Supplemental Figure S1

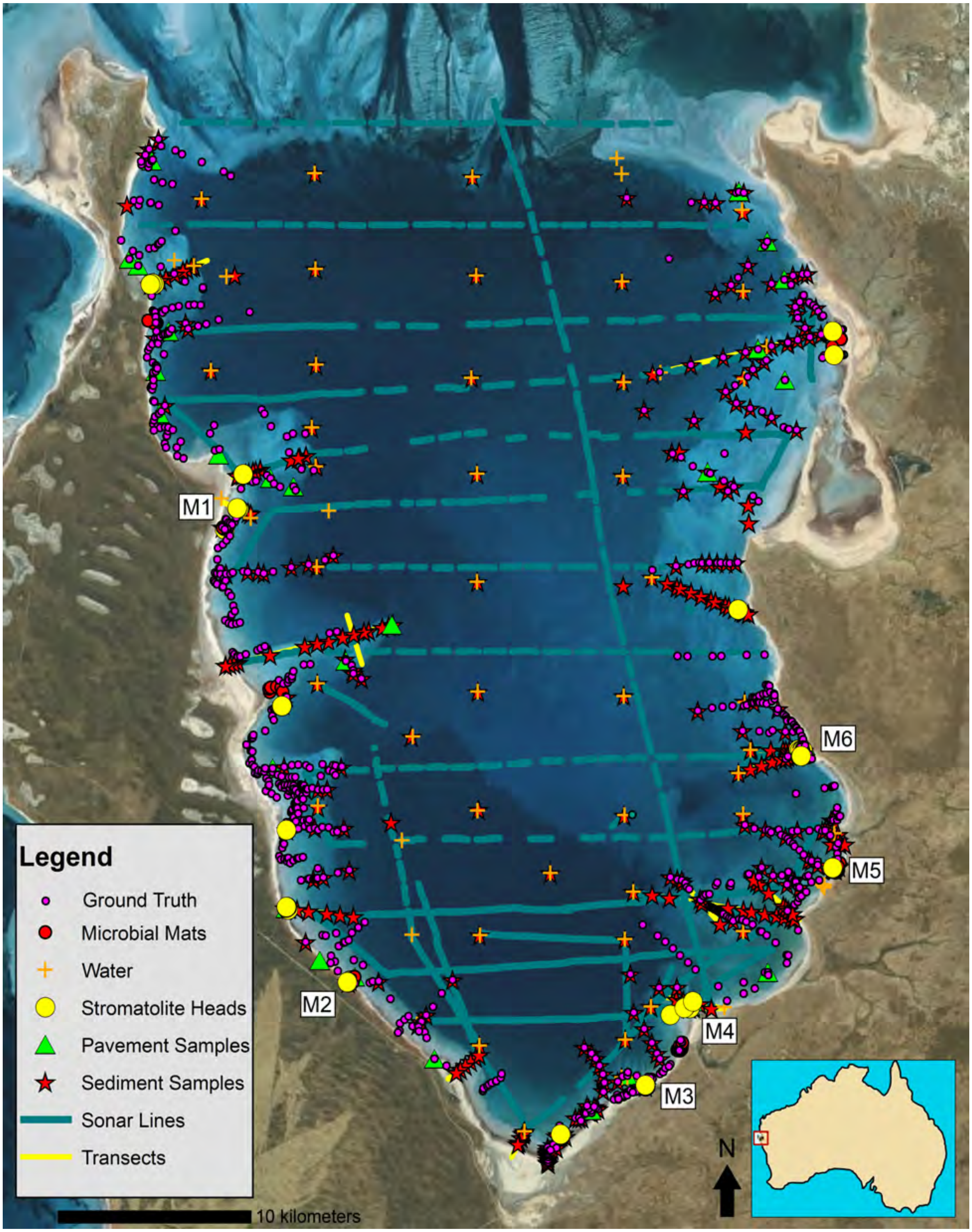

Supplementary Figure S1. Map showing ground-truth points for this study. Sample collection included 45 stromatolite heads, 84 microbial mats, 150 sediment and 50 water samples. Single beam sonar data collected throughout the pool provided ground truth data for the bathymetry map in Figure 1. Detailed facies maps are beyond the scope of this synthesis paper, but will be presented in subsequent publications. M1-M6 indicate samples for molecular analyses from stromatolite heads (see Fig. 3, SupplementaryTable S3, Fig. S5; M1: Spaven; M2: Booldah; M3: F-Play; M4: F-T7; M5: F-Goat; M6: Carbla). Map created in ArcGIS; Basemap sources: Esri, DigitalGlobe, Earthstar Geographics, CNES/Airbus DS, GeoEye, USDA FSA, USGS, Getmapping, Aerogrid, IGN, IGP, swisstopo, and the GIS User Community.

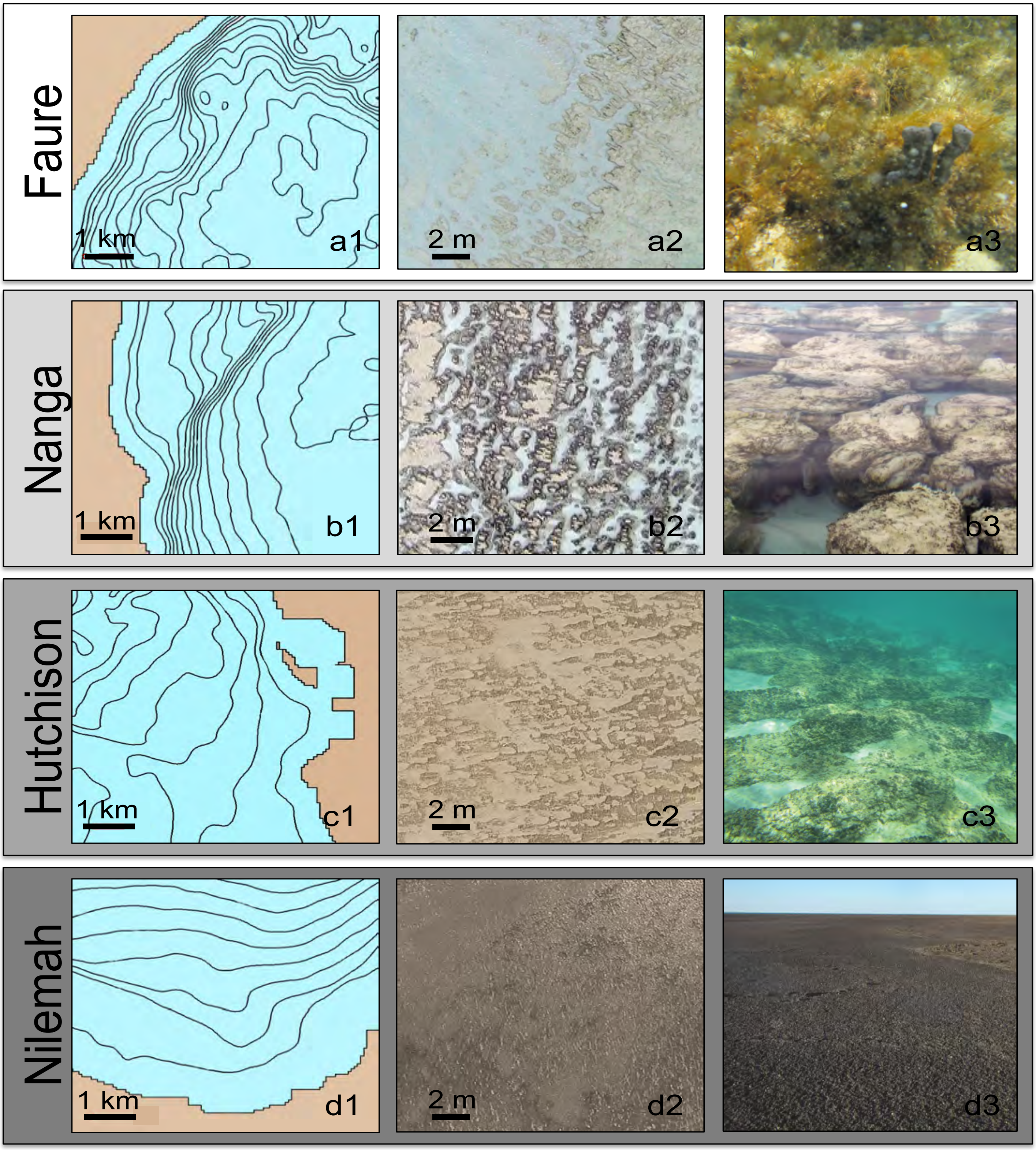

Supplementary Figure S2. Characteristic features of the four Provinces not shown in Fig. 2. Column 1 shows depth contours extracted from the bathymetry map (Fig. 1, boxes outlined in black); see text for discussion of physiography. Representative structures for each Province are as follows: Faure – eroded stromatolites and ribbon reefs composed of rock rubble; Nanga – tabular structures comprised of amalgamated stromatolites; Nilemah – continuous sheet mats; Hutchison – bands of elongate clustered stromatolites; Column 2 shows an example of sub-centimeter scale 2D imagery collected via UAV platform and processed using Fluid Lensing (see SI methods). Field photographs in Column 3 shows representative structures as follows: a3, an eroded stromatolite (~25 cm tall), encrusted with sponges, macroalgae, and mussels; b3, amalgamated tables ~ 50 cm tall; c3, elongate clustered stromatolites ~20 cm high; d3, continuous sheet of flat pustular mat.

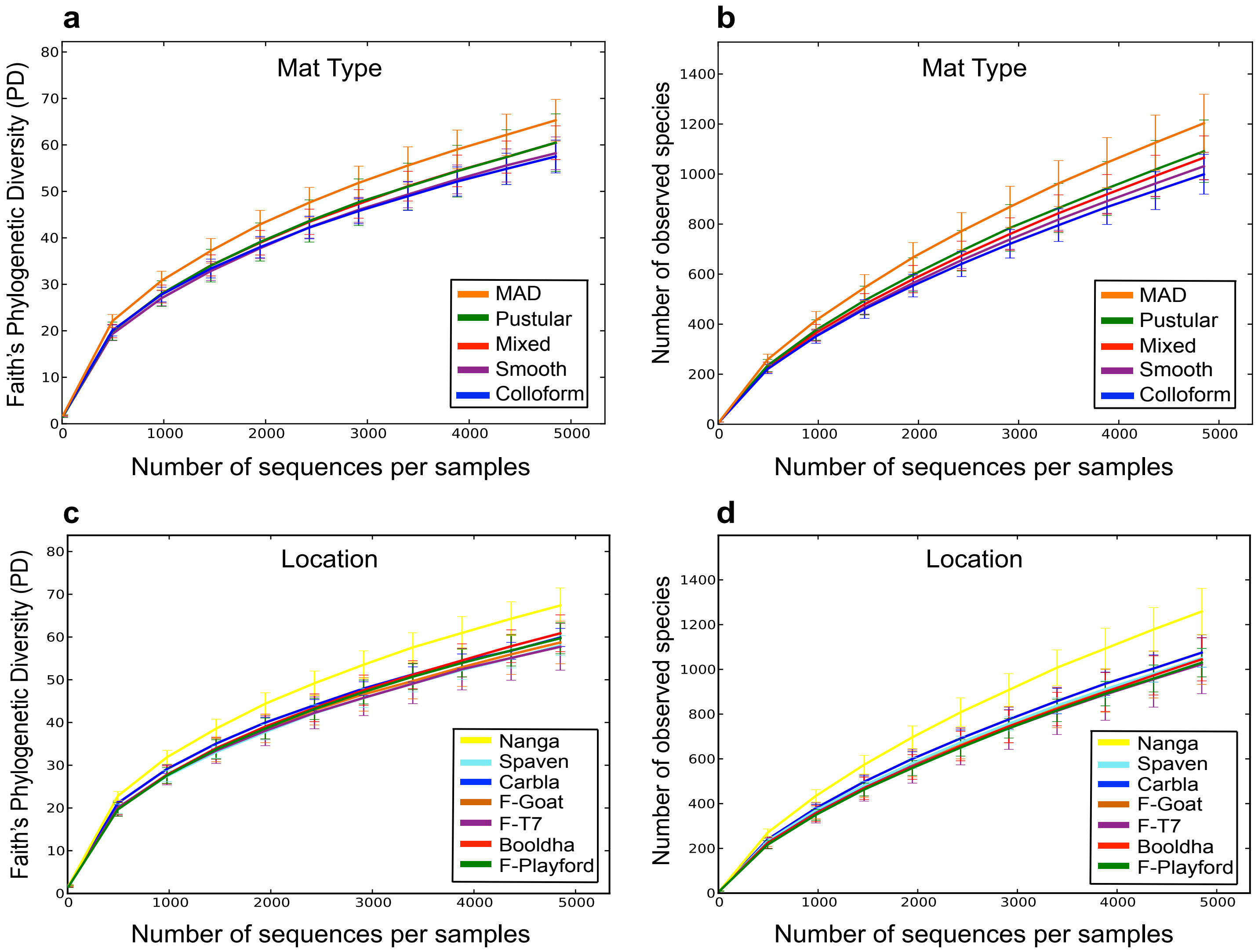

Supplementary Figure S3. Rarefaction curves according to different mat types and location. (a - b) Rarefaction curves were generated for each of the different mat types, as measured using Faith's Phylogenetic Diversity (PD) estimator (a) or as computed by observed species (b). (c - d) The rarefaction curves for both PD (c) and observed species (d) metrics, indicated a significant difference between mats based on geographical location. All rarefaction curves were obtained at a depth of 4,853 sequences per sample. Error bars represent standard error of the mean. Sample locations are shown in Supplementary Fig. S1.

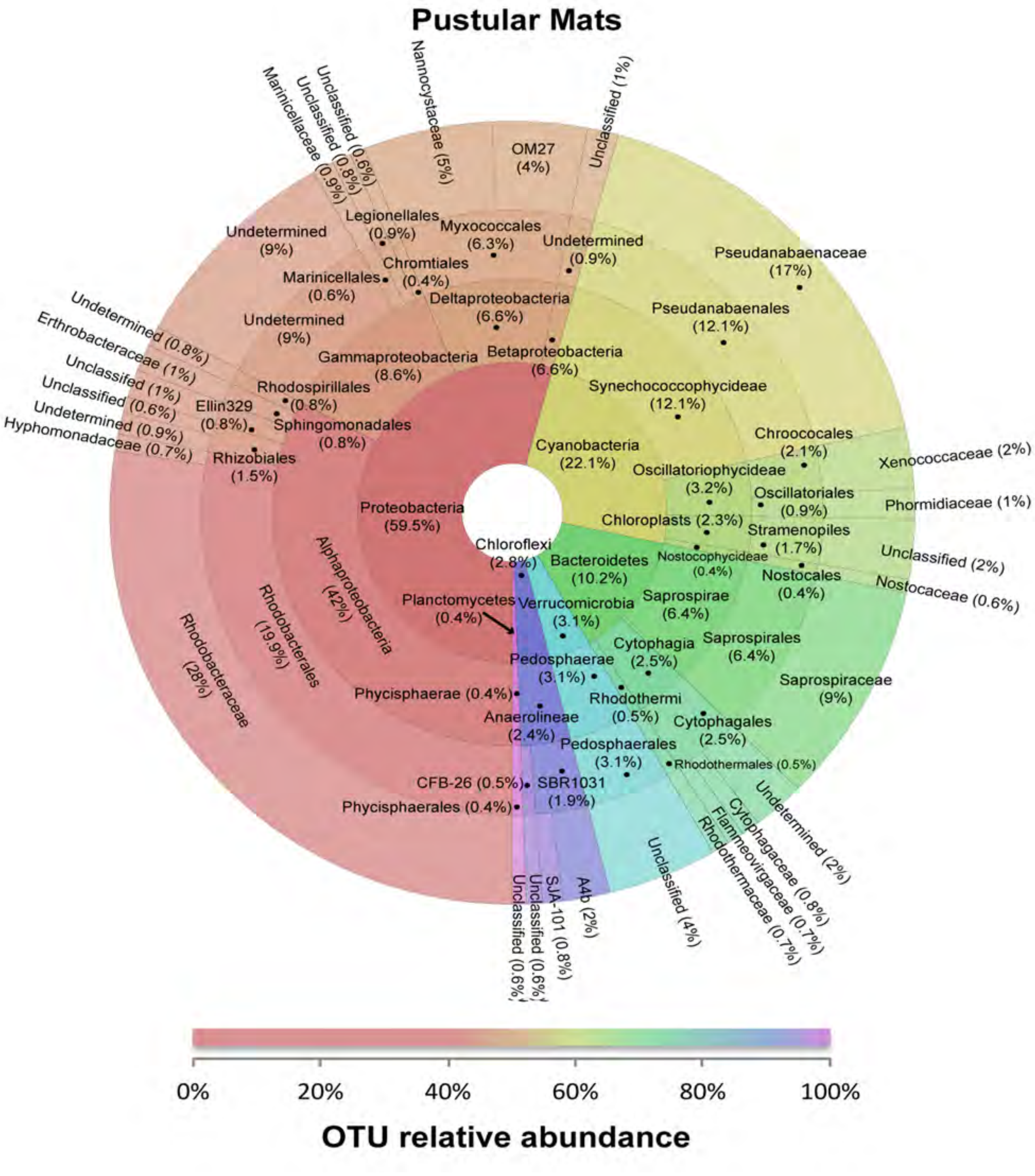

Supplementary Figure S4. Taxonomic abundance and diversity of the lithifying pustular mats. Krona plot visualizing the taxonomic hierarchies of the microbial mats with each ring representing a different classification level and different color. The inner ring represents the different phyla associated with the mats, and as the plot progresses outwards there is an increasing taxonomic resolution for each ring (i.e., class, order, family respectively). Those taxa at all levels comprising less than 0.4% of the total OTUs were not included in the plot.

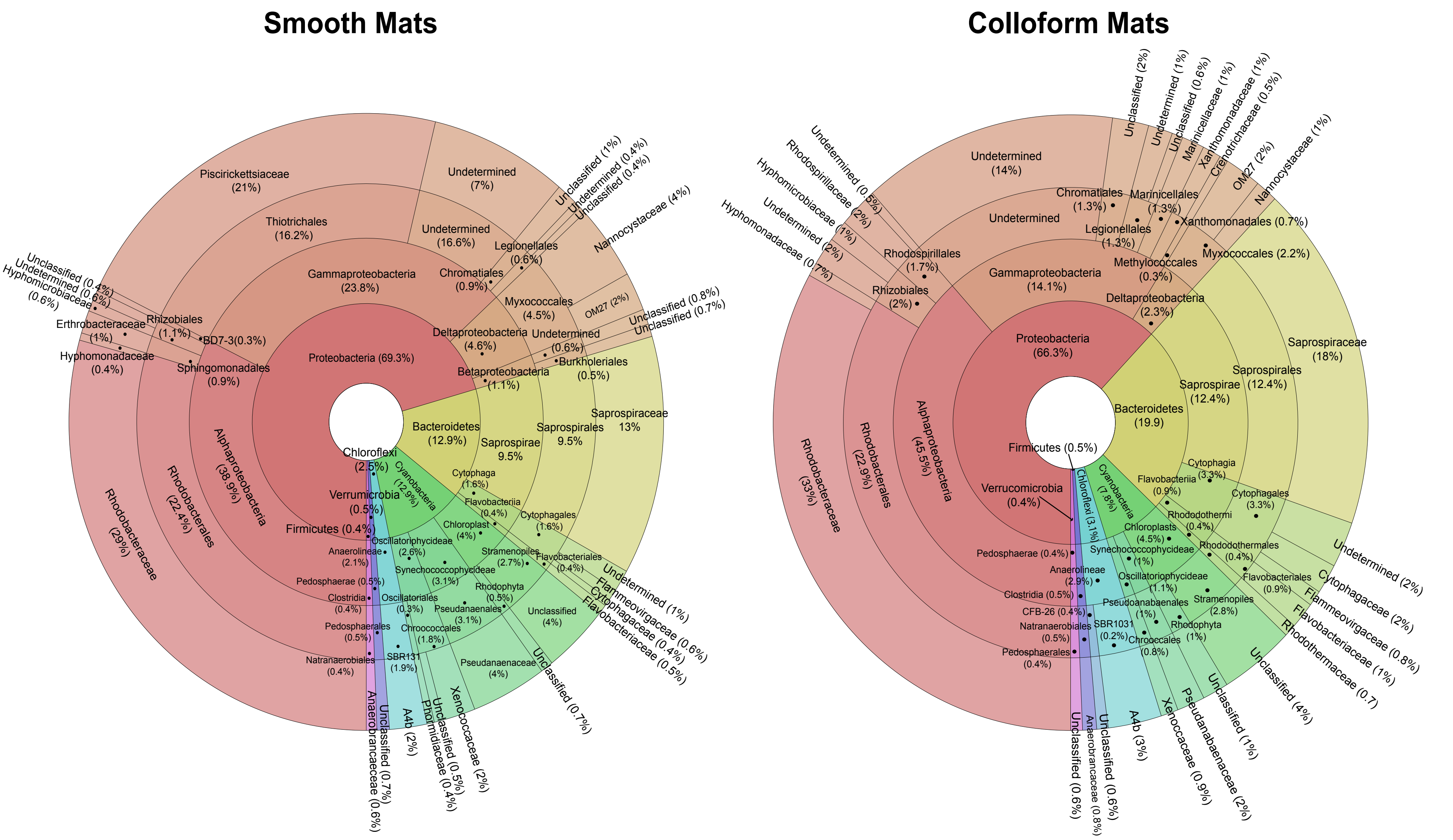

Supplementary Figure S5. Taxonomic abundance and diversity of the lithifying smooth and colloform mats. Krona plot visualizing the taxonomic hierarchies of the microbial mats with each ring representing a different classification level and color. The inner ring represents the different phyla associated with the mats, and as the plot progresses outwards there is an increasing taxonomic resolution for each ring (i.e., class, order, family respectively). Those taxa at all levels comprising less than 0.4% of the total OTUs were not included in the plot.

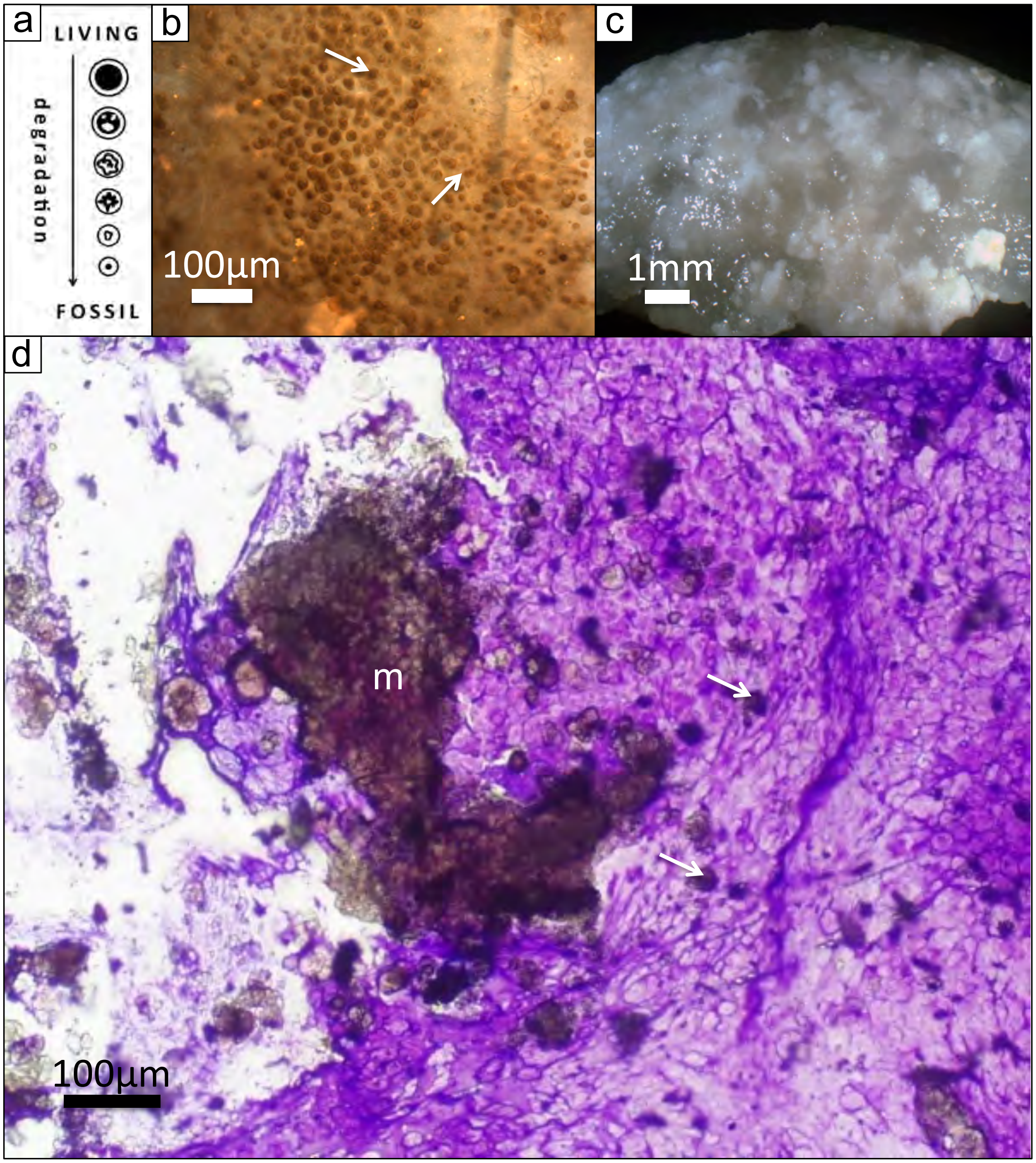

Supplementary Figure S6. **Calcification of the coccoid cyanobacterium *Entophysalis*. a**, Schematic cartoon showing the degradation of *Entophysalis* cells, after Golubic and Hofmann (1976). **b**, Photomicrograph showing healthy live *Entophysalis* cells; brown dots (arrows) are cells within thick polysaccharide-rich sheaths. **c**, Degrading eroded *Entophysalis* pustule; the clear gel is organic matter, the white is micritic precipitate. **d**, Thin section photomicrograph of pustule shown in (**c**); *Entophysalis* cells, stained purple with methylene blue, are being replaced by microcrystalline carbonate, micrite (m, arrows).
